# Validation of a rectal cancer outcome prediction model with a cohort of Chinese patients

**DOI:** 10.18632/oncotarget.5195

**Published:** 2015-09-18

**Authors:** Lijun Shen, Johan van Soest, Jiazhou Wang, Jialu Yu, Weigang Hu, Yutao U. T. Gong, Vincenzo Valentini, Ying Xiao, Andre Dekker, Zhen Zhang

**Affiliations:** ^1^ Department of Radiation Oncology, Fudan University Shanghai Cancer Center, Department of Oncology, Shanghai Medical College, Fudan University, Shanghai, China; ^2^ Department of Radiation Oncology (MAASTRO), GROW School for Oncology and Developmental Biology, Maastricht University Medical Centre, Maastricht, The Netherlands; ^3^ Department of Radiation Oncology, University of Pennsylvania, Philadelphia, PA, USA; ^4^ Department of Radiation Oncology, Università Cattolica S Cuore, Rome, Italy

**Keywords:** rectal cancer, preoperative chemoradiation, external validation, nomogram

## Abstract

The risk of local recurrence (LR), distant metastases (DM) and overall survival (OS) of locally advanced rectal cancer after preoperative chemoradiation can be estimated by prediction models and visualized using nomograms, which have been trained and validated in European clinical trial populations. Data of 277 consecutive locally advanced rectal adenocarcinoma patients treated with preoperative chemoradiation and surgery from Shanghai Cancer Center, were retrospectively collected and used for external validation. Concordance index (C-index) and calibration curves were used to assess the performance of the previously developed prediction models in this routine clinical validation population. The C-index for the published prediction models was 0.72 ± 0.079, 0.75 ± 0.043 and 0.72 ± 0.089 in predicting 2-year LR, DM and OS in the Chinese population, respectively. Kaplan-Meier curves indicated good discriminating performance regarding LR, but could not convincingly discriminate a low-risk and medium-risk group for distant control and OS. Calibration curves showed a trend of underestimation of local and distant control, as well as OS in the observed data compared with the estimates predicted by the model.

In conclusion, we externally validated three models for predicting 2-year LR, DM and OS of locally advanced rectal cancer patients who underwent preoperative chemoradiation and curative surgery with good discrimination in a single Chinese cohort. However, the model overestimated the local control rate compared to observations in the clinical cohort. Validation in other clinical cohorts and optimization of the prediction model, perhaps by including additional prognostic factors, may enhance model validity and its applicability for personalized treatment of locally advanced rectal cancer.

## INTRODUCTION

Colorectal cancer (CRC) is one of the three leading causes of cancer mortality worldwide and approximately one-third of the cancers arise in the rectum. The annual incidence of rectal cancer in China exceeds 200,000 cases and about 40% patients have stage III disease at the time of diagnosis. For locally advanced rectal cancer, preoperative radiotherapy combined with a fluoropyrimidine followed by total mesorectal excision (TME) is the current treatment standard [1]. However, the improvement in locoregional control observed in several randomized trials has not translated into improved survival [2–4]; the development of distant metastases is now the predominant cause of failure in locally advanced rectal cancer [5].

As many as 20% of patients may have a complete pathologic response after preoperative chemoradiation and have a very good prognosis, but the remainder of the patients have a higher risk for local recurrence and/or distant metastasis after treatment [6, 7]. We believe that rectal cancer treatment strategies should be personalized according to the expected outcome. Rectal cancer patients, for whom a poor outcome is expected, may benefit from intensified local or systemic treatment. In contrast, patients with an expected pathologic complete response (pCR) after preoperative chemoradiation may be considered for organ-preserving nonsurgical treatment strategies such as wait-and-see [8–11].

To personalize treatment, validation of prediction models is needed to create an evidence base for treatment decisions [12, 13]. Several nomograms for predicting follow-up outcome for colorectal cancer have been proposed, but models for locally advanced rectal cancer are scarce [14–16]. Valentini et al [17] developed prediction models (visualized using nomograms) for locally advanced rectal cancer patients treated with long-course chemoradiation (CRT) followed by surgery, based on data from large randomized trials. The discriminative capability of these nomograms was assessed during external validation, and was determined by measuring the concordance index (C-index) of local recurrence, distant metastases and overall survival (0.68, 0.73 and 0.70 respectively) after 5 years of follow-up. As this study performed an external validation on clinical trial data, it is unknown whether the findings are generalizable to an Asian routine patient population.

The aim of this study is to test the hypothesis that the published model to classify the probability of survival for locally advanced rectal cancer, developed by Valentini et al., is generalizable to a routine clinical dataset from an Asian cancer center.

## RESULTS

### Patient characteristics

The distribution of clinicopathologic characteristics between the European training cohort and the Chinese clinical validation cohort were shown in Table 1. The median age of the validation cohort was 56 years old. The median follow-up was 26 months (ranging from 3 to 87 months). Of the 277 patients, 20 (7.2%) patients developed local recurrence, 57 (20.6%) patients developed distant metastasis and 42 (15.2%) patients died during follow-up. There were significant distribution differences between two cohorts except the sex distribution.

**Table 1:** Patient characteristics of the European training (*N* = 2795) and the current clinical routine validation (*N* = 277) cohorts

Variable	Training cohort (*N* = 2235)	Current validation cohort (*N* = 277)	*p*-value
Sex			0.780
Male	1575 (70.5)	198 (71.5)	
Female	660 (29.5)	79 (28.5)	
Age, years			<0.001
≤49	290 (13.0)	107 (38.6)	
50–59	607 (27.2)	78 (28.2)	
60–69	917 (41.0)	69 (24.9)	
≥70	421 (18.8)	23 (8.3)	
Tumor location			<0.001
Low	786 (35.2)	116 (41.9)	
Mid	1146 (51.3)	158 (57.0)	
High	303 (13.6)	3 (1.1)	
cT stage			<0.001^*^
2	18 (6.1)	9 (3.2)	
3	1887 (84.4)	231 (83.4)	
4	193 (8.6)	37 (13.4)	
Treatments			
Radiotherapy dose, Gy			<0.001
<45^Ɨ^	95 (4.3)	20 (7.2)	
45	1558 (69.7)	39 (14.1)	
>45	582 (26.0)	218 (78.7)	
Concomitant chemotherapy			<0.001
No	862 (38.6)	5 (1.8)	
Yes	1373 (61.4)	272 (98.2)	
Surgery procedure			<0.001
LAR	1373 (61.4)	100 (36.1)	
APR	862 (38.6)	177 (63.9)	
Adjuvant chemotherapy			<0.001
No	836 (37.4)	15 (5.4)	
Yes	1399 (62.6)	262 (94.6)	
ypT stage			<0.001
0	205 (9.2)	64 (23.1)	
1–2	810 (36.2)	88 (31.8)	
3	1163 (52.0)	119 (43.0)	
4	57 (2.6)	6 (2.2)	
ypN stage			0.035
0	1551 (69.4)	175 (63.2)	
1–2	684 (30.6)	102 (36.8)	
median follow-up time	75 months	26 months	-
2y local control rate	92.0%	92.4%	0.708
2y distant control rate	77.8%	79.8%	0.431
2y overall survival	88.3%	93.7%	0.833

Values in brackets are intra-variable categorical percentages

* Fisher's exact test was used for cT stage.

Ɨ Patients receiving < 45 Gy were all receiving long-course chemoradiation but not 25 Gy in 5 fractions.

### Nomogram validation

When re-validating the previous prediction models from the training cohort of the original paper for at 2 years of follow-up, we found C-indexes of 0.72 ± 0.021, 0.73 ± 0.013 and 0.68 ± 0.018 for the prediction of local recurrence, distant metastasis and overall survival, respectively. The evaluation in the Chinese clinical routine population achieved C-indexes of 0.72 ± 0.079, 0.75 ± 0.043 and 0.72 ± 0.089, respectively.

**Figure 1:**
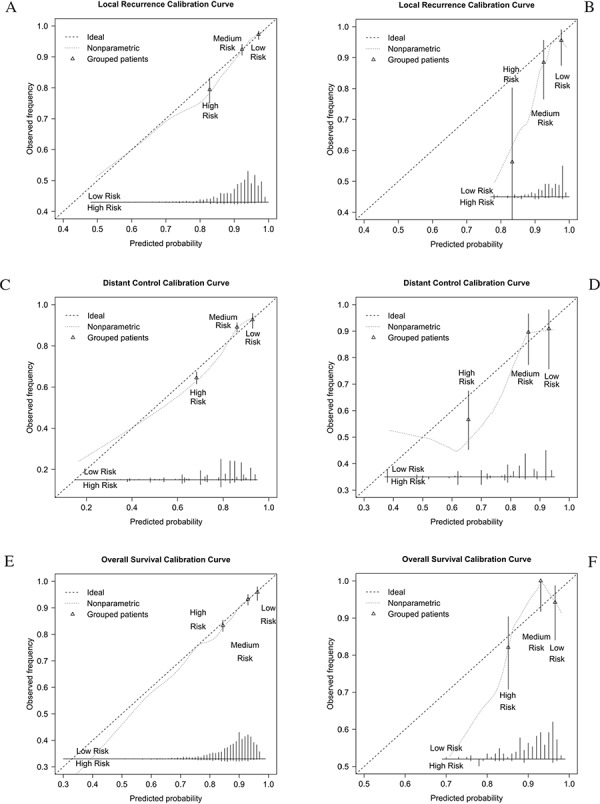
Calibration curves for prediction models in European training cohort and Chinese validation cohort **A.** Calibration curve for local control in training cohort. **B.** Calibration curve for local control in current validation cohort. **C.** Calibration curve for distant control in training cohort. **D.** Calibration curve for distant control in current validation cohort. **E.** Calibration curve for overall survival in training cohort. **F.** Calibration curve for overall survival in current validation cohort.

Figure 1 showed the calibration curves comparing the observed outcomes with the predicted LR, DM and OS probabilities in the original training cohort and the current validation cohort. Calibration curves in original training cohort suggested that the prediction models were well calibrated (Fig. 1A, 1C and 1E). Although the trend of observed incidence was lower than the predicted incidence in Chinese cohort, calibration curves were not different from ideal since the error bars touched the ideal line. The bars represent 95% confidence interval suggesting that we are for 95% sure that calibration group is similar to observation group. This means the nomograms have a trend to overestimate the probabilities of local and distant control, as well as overall survival in the validation population, especially in the high risk group.

**Figure 2:**
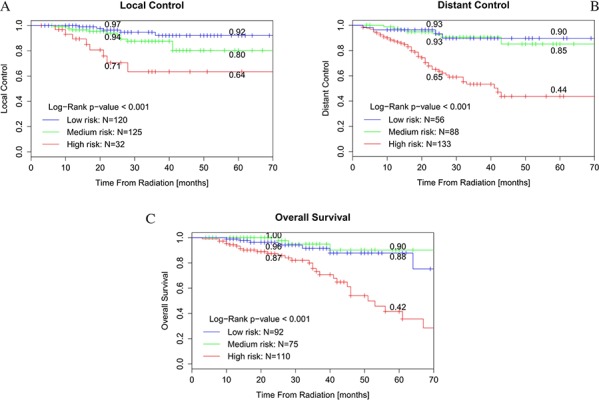
Kaplan-Meier curves for patients stratified by nomogram-predicted survival **A.** Kaplan-Meier curve for patients stratified by local recurrence risk of nomogram-predicted survival. **B.** Kaplan-Meier curve for patients stratified by distant metastasis risk of nomogram-predicted survival. **C.** Kaplan-Meier curve for patients stratified by death risk of nomogram-predicted survival.

Figure 2 showed the Kaplan-Meier curves stratifying patients into low, medium or high risk groups based on predicted outcome. This figure showed that the local control prediction model had discriminative power to stratify three risk groups (high, medium, low; *p* = 0.002), but it should be noticed that only 32 patients were assigned to the high risk group. The distant control and overall survival prediction models were able to significantly discriminate between the high-risk group and the medium- or low-risk groups (*p* < 0.001 and *p* < 0.001, respectively) but couldn't discriminate between medium- and low-risk groups.

## DISCUSSION

Nomograms as a rapid learning method and decision-support system have been developed for several types of cancer [18–20] and radiation oncology [13]. One significant advantage of nomograms is their ability to easily deduce a (survival or curative effect) probability for individual patients. In locally advanced rectal cancer, the prediction models, and subsequent nomograms, developed by Valentini et al. appeared to be an easy-to-use tool to stratify patients into three risk groups for local recurrence, distant metastases, and overall survival. It should be noticed that these models were trained and validated on European clinical trial populations; their generalization to the routine hospital population is unknown. It' necessary to validate in routine clinical setting to make sure models are actually performing well. We can try to optimize models with more features further before, however the question will be how generalizable they are. As to our knowledge, this is the first study validating these prediction models in a routine clinical, non-European patient population. Therefore, further external validation of these prediction models in other independent (routine clinical) datasets should be performed, since differences in population distribution and treatment may influence the accuracy and calibration of the model.

In our study, there are some differences in the distribution of clinicopathologic features between European clinical trial patients (training set) and this Chinese routine clinical cohort (Table 1). Firstly, patients in our cohort are younger than in the training set. Secondly, most cancers are located in the low to middle distance from the anal verge in our cohort and there is a higher abdominal-perineal resection (APR) surgery rate. This might be due to European has a taller body shape and higher peritoneal reflection than Asian population, and a decision bias from surgeons. Thirdly, radiation with more than 45Gy is more common in Chinese clinical cohort and there is a relatively higher ypT0 rate. The impact of an uneven distribution may lead to the underestimation of the probabilities of relapse and survival in the validation population.

**Figure 3:**
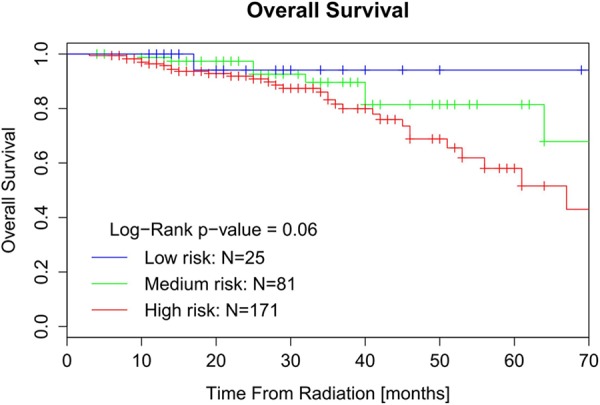
Kaplan-Meier curve of OS for patients stratified by ‘tailored’ cut-off value

We observed a good discriminative capability of the prediction models with a C-index of 0.72 ± 0.079, 0.75 ± 0.043 and 0.72 ± 0.089 for 2-year local recurrence, distant metastases and overall survival, respectively. Kaplan-Meier curves with stratification based on the predicted score indicated a good stratificational performance of local control in the clinical cohort, however, couldn't stratify low-risk and medium-risk groups well in distant control and overall survival. One important reason is that the number of patients in these risk groups is small in comparison to the original validation. The cut-off ranges of different risk groups in the nomograms are not optimized for this dataset and therefore introduce variation in risk group sizes in the clinical cohort. Since cut-off value is relatively subjective in different studies, we choose a ‘tailored’ cut-off to see if the model works well. After we redistributed the patients into different risk groups (the proportion approximately equals to European training data) by new cut-off value, the discrimination of low-risk and medium-risk groups seems better in Kaplan-Meier curve of OS (Figure 3). Therefore, not only the proper size of the population but also the ‘tailored’ cut-off values are important for the application of nomograms.

Several other limitations have to be taken into account in this study. Since preoperative chemoradiation for locally advanced rectal cancer in China started after the publication of several important randomized clinical trials, the follow-up time in the validation cohort was not long enough (median follow-up was 26 months and 23 of 277 patients lost to follow-up). Morever, our data were from a single institution with small sample size which may not be representative of rest of the Chinese population. More patients from multicenters are needed to enlarge the validation group. Furthermore, the current validation was based on 2-year follow-up, where the original prediction models were validated on 5-year follow-up. Although we used the adjusted baseline hazard, the original development (and variable selection) was based on 5-year follow up, and may have resulted in some differences. Nonetheless, we have shown that the prediction models still hold discriminative power when applied to a 2-year follow-up dataset.

In this retrospective study, 16 patients whose adjuvant chemotherapy information or pathological results were not available were excluded for validation, introducing a possible selection bias. Moreover, in the original trial datasets, local recurrence was diagnosed by histology, and distant metastasis by at least two imaging exams. These rules were not as strictly applied in our Chinese clinical routine validation cohort. This could cause an overestimation of relapse compared to the original trial population.

Given these limitations, the nomograms derived from European clinical trial population might not be ideally applicable to a routine Chinese population, however, they would work better with tailored cut-off values. Furthermore, as this is a validation based on routine clinical data, this validation better resembles the noise in data (incomplete data, other guidelines/policies) to be expected when applying a prediction model in the clinical setting [21]. Future work including Chinese and other clinical care populations in the training set are expected to improve its calibration as well as generalizability to the clinical care situation. As our understanding of the progress of rectal cancer, more specific clinical, pathologic, and biologic factors can be incorporated to refine this predictive model.

## MATERIALS AND METHODS

### Patients

Between March 2006 and December 2012, a consecutive series of 338 patients with MRI/CT-staged locally advanced (cT3–4 and/or cN1–2) rectal adenocarcinoma underwent long-course conventional chemoradiation (total dose was between 45–55Gy and mean dose was 50Gy) in daily fraction from Monday to Friday with a concomitant 5-fluorouracil-based chemotherapy and surgery in Shanghai Cancer Center. The patient records were retrospectively extracted from the clinical databases including electronic medical record and treatment planning system. All patients underwent a physical examination before neoadjuvant therapy, including digital rectal examination, and flexible endoscopy; computed tomographic (CT) scans of the chest, abdomen; and CT and/or magnetic resonance imaging (MRI) of the pelvis. Sixty-one patients were excluded for the following various reasons. Twelve patients were diagnosed with a non–skin cancer within 5 years of the diagnosis of rectal cancer. Twenty-seven patients did not undergo radical rectal resection and 2 patients did not complete radiation. Four patients had a metastatic disease before or at the time of surgery. For 16 patients the adjuvant chemotherapy information or pathological results were not available. Finally, a total of 277 patients with complete clinicopathological information were included in this study.

Patients were followed-up every 3–6 months during the first 2 years, every 6 months in the later 3 years, and after 5 years only once every year. Follow-up evaluation consisted of physical examination, imaging examinations, endoscopic study and laboratory examination. Local relapse was defined as tumor recurrence within the pelvis while distant metastasis was defined as out of the pelvis. All relapse cases were diagnosed either by histology or imaging exams. Overall survival was defined as the time difference between date of diagnosis and death from any cause in this study. Recurrence-free survival and metastases-free survival were defined as the time from the start of RT to local recurrence or distant metastasis. The protocol was approved by the hospital's Medical Ethics Committee.

### Statistical analysis

The variables required for the nomograms were gender, age, clinical tumor stage, radiotherapy dose, concomitant chemotherapy, surgery procedure, pathological tumor stage, pathological nodal stage and adjuvant chemotherapy. For each patient, the 2-year predicted probability of local control, distant control and overall survival was calculated using the previously published prediction model [17]. The model we used in this manuscript was come from the training cohort of the original paper. We used the same cohort to calculate the prediction performances for training dataset and used the full routine clinical dataset as validation dataset. In this previous publication, the authors trained a Cox proportional hazards model, and validated the binary outcomes for local recurrence, distant metastasis and overall survival at 5-years of follow-up. This means that the baseline hazard for a follow-up of 5 years was used. In this study, we evaluated the outcomes at 2 years of follow-up. Therefore, we used the baseline hazard for a follow-up of 2 years.

For the nomograms, this means that the sum of scores (the sum of all scores per prediction parameter) stays the same, but the probability related to the sum of scores value changed. To make a fair comparison, we recalculated the concordance index (C-index) on the external validation dataset used by Valentini et al. The C-index is defined as the proportion of all usable patient pairs in which the predictions and outcomes are concordant. The C-index measures predictive information derived from a set of predictor variables in a model. C-index is identical to the area under a “receiver operating characteristic” (ROC) curve in diagnostic discrimination. As we use binary value to defined patient outcome, it was equal to AUC in this manuscript. [22]. This AUC value represents the ability of a model to assign a higher probability to positive outcomes (e.g. survival). Calibration refers to the agreement between observed outcomes and predictions. Perfect predictions should be on the 45-degree line in calibration curve (the ideal calibration) [22]. Using the given cut-off values (that is, for local recurrence, the two probability thresholds were 8% and 20% while for distant metastasis and overall survival, the thresholds were 15% and 25%), patients were grouped in good, medium and poor prognosis groups [17].

The clinicopathologic characteristics of modeling and validation cohorts were analyzed by chi-square test or Fisher's exact test. Clinical survival outcomes were assessed using the Kaplan-Meier survival curves, and prognostic groups were compared with the log-rank test implemented in SPSS version 18.0 (SPSS, Chicago IL). A two-sided *p*-value of less than 0.05 was considered statistically significant.

The predicted and observed outcomes were compared using the C-index for discrimination performance assessment. To reduce selection bias and improve validation reliability, we bootstrapped (*R* = 1000) the predicted and observed outcomes and calculated the C-index for each bootstrap sample. Afterwards, we used the mean C-index from all bootstrap samples as the final C-index for our validation. Calibration curves were applied to assess the agreement between observed outcomes and predictions [23]. The C-index and calibration curves were implemented in Matlab version 7.1 (MathWorks, Natick, MA).

## Abbreviations

LR: local recurrence
DM: distant metastases
OS: overall survival
CRC: colorectal cancer
TME: total mesorectal excision
pCR: pathologic complete response
CT: computed tomographic
MRI: magnetic resonance imaging
C-index: concordance index
AUC: area under the curve
